# A Rare Presentation of Delusional Parasitosis With Koro-Like Syndrome

**DOI:** 10.7759/cureus.59946

**Published:** 2024-05-09

**Authors:** Nayan Sinha, Sneha B Suresh, Pradeep S Patil, Navaneetha Unni

**Affiliations:** 1 Psychiatry, Jawaharlal Nehru Medical College, Datta Meghe Institute of Higher Education and Research, Wardha, IND

**Keywords:** somatic delusion, delusional parasitosis, culture-bound syndrome, koro, delusion

## Abstract

Delusional parasitosis (DP) with Koro-like syndrome poses a complex clinical challenge, demanding a comprehensive and empathetic approach from healthcare professionals. This exceptional combination of fixed beliefs about infestation and experiences of genital retraction can profoundly impact patients' well-being and daily functioning. The associated stigma and misconceptions further compound the difficulties faced by individuals struggling with these co-occurring conditions. Given the rarity of encountering both conditions simultaneously, navigating the diagnosis and treatment of delusional parasitosis with Koro-like syndrome requires a thorough understanding of its multifaceted nature. Embracing a holistic strategy encompassing psychoeducation, psychotherapy, and pharmacological interventions is essential for effectively addressing these dual conditions.

## Introduction

Delusional disorder involves the persistence of a delusion or a set of related delusions lasting at least three months, typically longer, without concurrent depressive, manic, or mixed mood episodes. The symptoms are not attributable to another disorder or disease, nor are they induced by substances or medications [1]. The persecutory subtype is frequently cited in Western literature, while delusional parasitosis is more commonly documented in Indian literature [2].

Delusional parasitosis (DP) is a persistent belief in the presence of tiny organisms on or within the skin, despite evidence to the contrary [3]. It manifests in two forms: primary, unrelated to other conditions, therefore fulfilling the criteria for persistent delusional disorder or somatic-type delusional disorder [4], and secondary, occurring alongside psychiatric or physical disorders, or induced by certain medications. DP commonly coexists with psychiatric conditions such as depression and schizophrenia, as well as physical ailments such as renal insufficiency, multiple sclerosis, diabetes mellitus, hepatitis, vitamin B12 deficiency, iron deficiency anemia, or leprosy. Medications such as amantadine and levodopa can also trigger DP [5-7]. Its symptoms include sensations resembling crawling or biting insects [8].

Koro syndrome is a psychiatric disorder characterized by an intense fear of genitalia retracting into the body, potentially leading to death. In males, this fear centers on the penis shrinking into the abdomen, while in females, it involves the vulva and breasts shrinking into the abdomen and chest [9]. It is categorized into two types: endemic and sporadic. The endemic type, prevalent in East and Southeast Asia, is culturally linked and rooted in historical literature or folklore [10]. It presents with classic symptoms of primary Koro, including severe anxiety and fear of death. On the other hand, sporadic Koro, also known as Koro-like syndrome, lacks cultural ties and can occur elsewhere. Symptoms, such as genital shrinking without a belief in imminent death, typically arise secondary to conditions such as schizophrenia, affective disorders, medical illnesses, or substance abuse [11].

Diagnosing Koro involves both psychological assessment and physical examination of the genital organs, the latter aimed at excluding physical conditions such as hypospadias or measurable, sustained genital retraction. The primary diagnostic criteria include patients reporting genital (e.g., penis) retraction despite objective evidence, followed by fear and anxiety [12,13].

There is limited literature available that describes delusions with a sexual theme. They are typically seen in patients with other psychotic disorders [2]. This case describes how a patient's beliefs, their interconnectedness, and the emergence of depressive symptoms are intricately intertwined.

## Case presentation

A 23-year-old unmarried male, pursuing a degree in Master of Science in Botany, belonging to a low socioeconomic status background, with a supportive family, with cluster C traits as per the Diagnostic and Statistical Manual of Mental Disorders, Fifth Edition (DSM-5) [14], and unremarkable family history, presented with a three-year long history of discomfort in the form of tingling sensations and numbness in his abdomen and chest region. He believed that he was infested with insects and started complaining of abnormal crawling sensations in his abdomen, which was spreading to his entire body. These perceptual disturbances (crawling sensations) were not prominent and were related to his fixed belief. Ambiguous regarding the nature of the insects, he considered them to be bacterial or viral in nature. The reported issues occurred concurrently with the COVID-19 pandemic, during which the patient expressed feeling apprehensive and concerned about the possibility of contracting the virus, either personally or within his family, despite having tested negative for it.

The patient also complained of loss of rigidity of his genitals and failure to maintain an erection. He believed that his penis was shrinking into his abdomen and had decreased in size over the past three years. There was also a loss of nocturnal penile tumescence in the past two months. He attributed these changes to the insects in his body but could not explain the mechanism behind them. The belief was held with conviction despite questioning and contrary evidence in the form of a physical examination of his genitals. Distressed due to his beliefs, he remained preoccupied with them most of the day. Despite consulting various physicians and following their recommended treatments, the patient's condition showed no signs of improvement. The patient did not have the records detailing the treatments received.

Two months ago, the patient vomited multiple times after eating homemade food, with vomit containing food particles and water. He described the consistency as resembling a spider web and felt it was filled with invisible insects. Following the vomiting episodes, he experienced restlessness, nausea, sweating, shivering, palpitations, moderate chest pain on both sides, breathlessness, and a sense of impending doom every 2-3 days, lasting for 15 minutes, for the past two months. He believed that the insects were causing his symptoms by obstructing his chest. He was observed to be consistently placing his hand over his chest area to alleviate the breathlessness and chest pain. Relatives noted his visible distress and frequent requests for investigations to confirm the presence of insects in his body. He was hospitalized a month ago after a similar episode of restlessness, where all his medical tests, the records of which are not available, yielded normal results. Following his hospital discharge, he sought advice from a psychiatrist who prescribed him a 20-day trial of tablet escitalopram 10 mg HS and tablet clonazepam 0.5 mg HS. While there was some improvement in his anxiety symptoms, his belief of infestation and genital retraction secondary to the infestation continued to persist firmly.

Despite repeated reassurances from doctors, the patient remained convinced that something was crawling and multiplying inside him, causing his various complaints. He rejected alternative explanations for his condition and insisted on more rigorous testing for his diagnosis. After not experiencing any relief in his complaints, he developed a persistent low mood over the past two months, believing he was dying as insects were gradually causing internal damage. He was stressed about his poor financial condition and how he was becoming a burden to his family by staying sick.

Additionally, he expressed a loss of interest in pleasurable activities such as watching television or engaging with family members. This also led to him skipping classes and missing college examinations last month due to him being preoccupied with health worries, causing significant socio-occupational impairment. Persistent thoughts about insects disrupted his sleep initiation and maintenance, while diminished appetite was attributed to vomiting whenever he ate, believed to be caused by the insects inside him.

Investigations were done for the patient to rule out any physical conditions that could lead to the patient's belief of infestation such as complete blood count, liver function test, kidney function test, thyroid profile, fasting blood sugar, serum vitamin B12, serum vitamin D, electrocardiogram, urine routine, and culture. They were within normal limits. The patient and his family did not give consent for neuroimaging due to financial constraints. His overall physical and systemic examination yielded normal results as well.

The patient was admitted and, after detailed evaluation, was diagnosed with delusional disorder, currently symptomatic, with erectile dysfunction, and depressive symptoms as per the International Classification of Diseases 11th Revision (ICD-11) [1]. The following diagnosis was made despite the presence of tactile hallucinations because as mentioned in DSM-5 and ICD-11, in delusional disorder, hallucinations, if present, are not prominent and are related to the delusional theme [1,14]. Treatment began with tablet risperidone 2 mg once at night (HS), gradually increased to 6 mg HS, and tablet trihexyphenidyl (THP) 2 mg added once a day (OD) as a precaution for extrapyramidal symptoms. Tablet propranolol 20 mg OD and tablet clonazepam 0.5 mg twice a day were given for his restlessness and sleep disturbances.

Significant improvement in his mental status examination and insight was observed within 10 days. Additionally, supportive psychotherapy aimed at reducing his beliefs and distress was given, focusing on establishing a strong and positive connection with the patient. Reassurance was provided that his distress was acknowledged as genuine, adopting a neutral approach regarding his belief about being infested by insects and genital retraction to avoid reinforcing the delusion. The patient had little knowledge regarding genital physiology. He was educated on the normal physiology of the genitalia, and myths related to genital retraction were debunked.

The patient also exhibited cluster C traits, indicative of avoidant personality. His complaints emerged during the pandemic, due to the fear of him or his family contracting the infection. Therefore, it was crucial to address this, coping strategies were taught to him, and cognitive behavioral therapy sessions were taken.

Gradually, the patient improved and was discharged in a stable condition. In subsequent follow-up sessions, notable improvement was observed in his condition, enabling the patient to return to his classes. He no longer exhibited any of his previous complaints and had gained insight into his condition. Tablet clonazepam was tapered and stopped. He was then maintained on tablet risperidone 6 mg HS plus THP 2 mg OD.

## Discussion

Our patient's unwavering conviction in his belief, despite contradicting evidence, indicated that it was indeed a delusion, upon which he was basing his actions of seeking multiple consultations [2,14]. The distress expressed by the patient stemmed from his belief, and there were no indications of a primary mood disorder or any other psychotic symptoms, thus making it a delusional disorder [2]. The treatment and management of delusional disorder and its associated symptoms require a mixture of medical, psychological, and social interventions. At present, there is limited literature available on comorbid delusional parasitosis and Koro or Koro-like syndrome. Such patients frequently consult several doctors hoping to find one who will trust them, underscoring the significance of nurturing a constructive bond between physicians and patients.

Diagnosing this condition poses significant challenges. Initially, we need to eliminate all dermatological conditions that could manifest similarly. Following this, a thorough evaluation is necessary to determine the type of delusional parasitosis present [8]. In primary cases, there are no apparent physical signs, and investigations typically yield normal results. However, secondary cases may exhibit signs and symptoms of various physical ailments contributing to delusional parasitosis [7]. Further complicating matters is the differentiation between delusional disorder and hallucinations. Our patient exhibited both the delusion of infestation and tactile hallucinations in the form of crawling sensations. According to DSM-5 and ICD-11 criteria, in delusional disorder, hallucinations, if present, are not prominent and are linked to the delusional theme, such as the sensation of being infested with insects associated with delusions of infestation [1,14]. Although our patient experienced crawling sensations, their prominence was overshadowed by his delusional belief.

Historically, pimozide was initially employed as the primary treatment for delusional parasitosis, although its side effects are considered less desirable when compared to second-generation (atypical) antipsychotic medications [15]. Risperidone, olanzapine, aripiprazole, paliperidone, ziprasidone, and quetiapine have all demonstrated efficacy when used alone for DP treatment. Additionally, serotonergic medications have been noted as effective in individual case studies. Among these options, risperidone, aripiprazole, and olanzapine are the most mentioned in the literature [16].

In Koro, the retraction of the penis is characterized as a dynamic event, often described as sudden and rapid. In contrast, in delusional disorders such as the somatic type, this process tends to be gradual or prolonged, typically involving distinct cognitive aspects [17]. Our patient exhibited symptoms indicative of Koro-like syndrome. His anxiety regarding genital retraction stemmed from a belief of infestation, triggering panic attacks and resulting in subsequent erectile dysfunction. It is crucial to thoroughly explore a patient's sexual history to understand how psychosexual dysfunction, feelings of guilt, or past trauma may contribute to the development of unusual perceptions regarding penile morphology [17]. The therapeutic approaches for this condition primarily focus on addressing the root psychiatric issues through medication and psychotherapeutic support, with outcomes varying [18].

However, the scarcity of literature and case reports presents a challenge in identifying the most effective pharmacological approach for addressing the co-occurring delusional parasitosis and Koro-like syndrome. Anxiolytics, antidepressants, sedatives, or antipsychotics might be recommended based on the patient's accompanying psychiatric conditions, if relevant, as improvement in these conditions often aligns with the alleviation of Koro symptoms [9]. These pharmacological interventions primarily aim at addressing the underlying psychiatric conditions. In cases such as this, where there is an underlying psychotic disorder, the treatment for sporadic Koro hinges on its cause and the effectiveness of antipsychotics in managing the related psychotic symptoms [19]. Psychotherapeutic support and psychoeducation play pivotal roles in addressing the psychosexual and psychiatric aspects of these conditions. Sensitively exploring the patient's underlying personality traits, their understanding of sexual organs, providing education on genital morphology, and addressing culturally ingrained myths are fundamental components of comprehensive psychotherapeutic approaches in these cases [10].

## Conclusions

Delusional parasitosis with Koro-like syndrome presents a unique set of challenges for both patients and healthcare professionals as it is uncommon to encounter both simultaneously. Considering the unique nature of this condition, management strategies have been customized to meet the individual needs of the patient. Such an approach, while beneficial, can also limit the ability to draw definitive conclusions about treatment efficacy since it is patient-centric. One significant point to note is that the stigmatization and misunderstanding surrounding this condition further exacerbate the challenges faced by patients. It is crucial for healthcare professionals to approach this condition with empathy and understanding, as well as to understand the complexities involved for effective diagnosis and treatment. Addressing the dual conditions required a comprehensive strategy involving a combination of pharmacotherapy, psychoeducation revolving around breaking cultural myths, and cognitive behavioral therapy to address the patient's underlying personality traits to prevent any relapse.
